# Skin Conductance Responses of Learner and Licensed Drivers During a Hazard Perception Task

**DOI:** 10.3389/fpsyg.2021.619104

**Published:** 2021-03-25

**Authors:** Theresa J. Chirles, Johnathon P. Ehsani, Neale Kinnear, Karen E. Seymour

**Affiliations:** ^1^Center for Injury Research and Prevention, Bloomberg School of Public Health, Health Policy and Management Department, Johns Hopkins University, Baltimore, MD, United States; ^2^Transportation Research Laboratory, Wokingham, United Kingdom; ^3^School of Medicine, Department of Psychiatry, Johns Hopkins University, Baltimore, MD, United States; ^4^Bloomberg School of Public Health, Department of Mental Health, Johns Hopkins University, Baltimore, MD, United States; ^5^National Institutes of Health, Center for Scientific Review, Bethesda, MD, United States

**Keywords:** electrodermal activity, autonomous vehicles, driving experience, hazard perception, young drivers

## Abstract

**Background**: While advanced driver assistance technologies have the potential to increase safety, there is concern that driver inattention resulting from overreliance on these features may result in crashes. Driver monitoring technologies to assess a driver’s state may be one solution. The purpose of this study was to replicate and extend the research on physiological responses to common driving hazards and examine how these may differ based on driving experience.

**Methods**: Learner and Licensed drivers viewed a Driving Hazard Perception Task while electrodermal activity (EDA) was measured. The task presented 30 Event (hazard develops) and 30 Non-Event (routine driving) videos. A skin conductance response (SCR) score was calculated for each participant based on the percentage of videos that elicited an SCR.

**Results**: Analysis of the SCR score during Event videos revealed a medium effect (*d* = 0.61) of group differences, whereby Licensed drivers were more likely to have an SCR than Learner drivers. Interaction effects revealed Licensed drivers were more likely to have an SCR earlier in the Event videos compared to the end, and the Learner drivers were more likely to have an SCR earlier in the Non-Event videos compared to the end.

**Conclusion**: Our results support the viability of using SCR during driving videos as a marker of hazard anticipation differing based on experience. The interaction effects may illustrate situational awareness in licensed drivers and deficiencies in sustained vigilance among learner drivers. The findings demand further examination if physiological measures are to be validated as a tool to inform driver potential performance in an increasingly automated driving environment.

## Introduction

Advanced driver assistance systems (ADAS) have the potential to drastically reduce vehicle crash injury and death but may be accompanied with possible setbacks. Recent experience demonstrates that overreliance on this technology poses a separate set of risks where drivers may be unable to regain vehicle control in situations of technology failure (Krompier, 2017; Vogelpohl et al., 2019). One approach to addressing this problem is to augment safety by monitoring driver state (e.g., drowsiness, workload, and levels of vigilance) by using physiological measurements (Balters and Steinert, 2017; Lohani et al., 2019), but a model to understand the complexity of the relationship between physiological measures, individual driver cognitive state, and the implications for driving behavior and performance is far from complete (Balters and Steinert, 2017).

One psychophysiological measure of autonomic arousal utilized to monitor driver state is electrodermal activity (EDA). EDA is a measure of neuronally mediated autonomic changes in the electrical properties of the skin, and has been shown to be a sensitive index of sympathetic nervous system activity (Braithwaite et al., 2013; Dawson et al., 2017). Tonic skin conductance levels and phasic skin conductance responses (SCR), are elements of EDA that have long been used in the driving literature to measure workload, risk of accident (Hulbert, 1957; Taylor, 1964; Helander, 1978), as well as levels of stress and tension (Michaels, 1960; Healey and Picard, 2005). A recent study by Darzi et al. (2018) found decreased tonic skin conductance levels to be indicative of sleep deprivation. However, if driver state monitoring is to become a successful intervention to facilitate the safe interplay of driver assistance technology and driver manual takeover, psychophysiological monitoring models must not only incorporate cognitive states but also how individual responses may vary based on experience (Collet and Musicant, 2019) and the acquisition of critical driving skills.

For example, a critical skill that develops with driving experience is hazard perception (Quimby et al., 1986; McKenna and Crick, 1991; Horswill and McKenna, 2004). Hazard perception is the learned ability to detect, predict, recognize, and respond to developing hazards (Horswill and McKenna, 2004; Wetton et al., 2011; Crundall et al., 2012; Crundall, 2016) and has been associated with crash risk (McKenna and Crick, 1991). Kinnear et al. (2013) found that compared to novice drivers, experienced drivers were twice as likely to demonstrate an SCR when watching videos containing a driving hazard. The videos used in this study were validated to distinguish between novice and experienced drivers as part of the development of the United Kingdom hazard perception test. The difference between novices and experienced drivers was in the period leading up to the hazardous event, termed the “anticipatory period.” Subsequent hazard perception and SCR research have found similar results (Tagliabue and Sarlo, 2015; Barnard and Chapman, 2016; Tagliabue et al., 2017) but have been conducted outside the United States.

A potential reason for these differing autonomic responses to driving hazards between novice and experienced drivers emerges from literature suggesting the role of somatic experience on decision-making. Specifically, evidence suggest that this learning not only occurs from explicit knowledge of reward/punishment schedules, but also from affect-based somatic signals (i.e., pulse rate blood flow, pupil response, etc.) experienced by the driver (Damasio, 1994; Phelps et al., 2014; Petracca, 2020). Known as the somatic marker hypothesis (Damasio, 1994), this theory has potential implications for novice vs. experienced drivers suggesting that prior positive or negative experience results in the formation of a gut feeling or “somatic marker” (i.e., a physiological response) which in turn biases the options available for decision-making when encountering a similar situation in the future. This “feeling-based” system for decision-making complements and operates in parallel to the rational decision-making process, which if it were operating in isolation would take too long to reach complex decisions. However, decision speed and accuracy could be ecologically viable if facilitated using feedback from the autonomic and the somatic nervous systems *via* the emotion circuitry in the brain (Bechara and Damasio, 2005). Taken together, these biologically based decision-making theories have relevance for driver assessment of hazards, a decision-making process which needs to occur rapidly. Drivers who have progressed past the novice (or learner) stage would have a larger library of experience to draw from, allowing their feeling-based appraisal to identify potential risks earlier, and bias a behavioral response to anticipate and avoid the impending hazard.

The purpose of this study was to replicate and extend research investigating measures of autonomic arousal (i.e., EDA) during the viewing of driving hazards for young drivers differing in experience levels in the United States context. As the stimuli developed for previous studies were from the United Kingdom, they could not be readily used in the United States context due to the differences in the driver position in the vehicle and the direction of travel lanes. Thus, we developed a novel Driving Hazard Perception Task (Ehsani et al., 2020) and measured SCR during videos where a hazard occurred (Event), and videos of routine driving (Non-Event). Videos were extracted from real-world driving captured as part of a large-scale naturalistic driving study. While driving simulators more closely mimic the on-road driving task, the focus here was autonomic responses to developing hazards, rather than driving task performance. Videos including naturally occurring cues therefore provided the stimuli necessary for this study. The use of videos for this purpose has been demonstrated previously (Kinnear et al., 2013) and is commonly used for hazard perception testing. We hypothesized that more experienced drivers (Licensed) would have a greater likelihood of SCR than Learner drivers during Event videos, and both Learner and Licensed drivers would have a greater likelihood of SCR during the Event videos compared to the Non-Event videos. By replicating and extending the literature supporting the finding of heightened autonomic arousal during hazard anticipation induced by experiential learning, we would be providing valuable information to the developers of driver state monitoring systems.

## Materials and Methods

### Participants

Participants were recruited through flyer, email, website announcements, and in person from the Baltimore metro region. To be included in the study, participants needed to be between the ages of 16–20 years and have a valid driver’s license or learner’s permit and speak English fluently. To be included in the Learner group, drivers held a valid learner’s permit and had driven less than 1,000 miles as assessed by self-reported mileage. To be included in the Licensed group, participants had a valid non-commercial driver’s license for a minimum of 2 years and had driven more than 3,000 miles in the past 12 months as assessed by self-reported annual mileage. Exclusionary criteria included: (1) a history of neurologic disorder (e.g., epilepsy, cerebral palsy, traumatic brain injury, and Tourette syndrome), (2) a history of visual impairment, (3) the inability to read English fluently, and (4) the presence of psychiatric illness or neurodevelopmental disorders assessed *via* The Mini International Neuropsychiatric Interview for Children and Adolescents (MINI-KID). All study procedures were approved by the Johns Hopkins Medicine Institutional Review Board.

### Study Visit

Participants came to a single study session after passing an initial phone screening interview. Participants were introduced to the physiological recording equipment after informed consent and eligibility for the study confirmed with the MINI-KID. After the physiological recording equipment was placed on the participant and the quality of the data collection was verified, a 2-min baseline measurement was collected (the participant sat quietly and was task free) before the commencement of The Driving Hazard Perception Task that included 60 30-s videos [30 Event videos (hazard develops) and 30 Non-Event videos (routine driving)]. The development and details about the task may be found in Ehsani et al. (2020) and in the Supplementary Material. After the task, the recording equipment was removed, and participants completed demographics and medication history questionnaires. The participants were provided with a $50 gift card as compensation.

### Calculation of SCR Score

**Figure 1:**
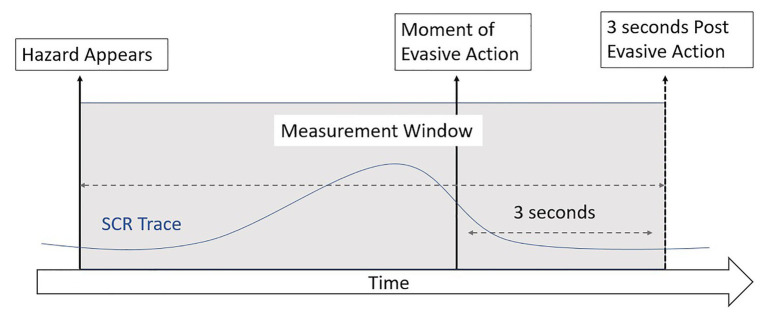
*Measurement windows* for event videos.

*Measurement windows* in the Event videos were defined from the first frame the hazard appeared on the screen to 3 s after the driver was required to perform an evasive action (see Figure 1). The window included the evasive action due to the delayed response of SCR (Braithwaite et al., 2013). These windows approximated the *anticipatory period* from the study of Kinnear et al. (2013). In Non-Event videos, non-hazardous occurrences were randomly selected from general driving clips with timing that corresponded to the event videos. Descriptions of the frames chosen to define the *measurement windows* are in Supplementary Table S1 (Supplementary Material). To avoid learning effects, the *Onset Time* of these *measurement windows* were staggered so that in the Event and Non-Event videos, *Early Onset videos* had the window at the beginning, *Middle Onset videos* had the window in the middle, and the *Late Onset videos* had the window toward the end of the video.

For an SCR to be included in the data, the phasic component increase of the EDA signal was required to be equal to or exceed 0.03 μS (Braithwaite et al., 2013), and the waveform onset was initiated within *measurement window*, and the peak was achieved within 10 s of the waveform onset. While Kinnear et al. (2013) used a 0.05 μS threshold, we opted to use the current acceptable threshold due to improvements in technology. These analyses were performed using *AcqKnowledge* Biopac Basic Scripting Software, 5.0, and the results were visually inspected for quality control. Participants were monitored during the data acquisition for behavior that might induce an SCR artifact (e.g., yawning, deep breaths, and body movement). There were no SCRs attributed to these artifacts during the *measurement windows*.

Repeating the method in Kinnear et al. (2013), we calculated an SCR score. This dependent measure reflects non-responses as well as responses. To calculate an *SCR Score*, the number of Event and Non-Event video clips where the participant exhibited an SCR response during the *measurement window* were summed. To do this, Event periods were coded (using Matlab) as a 0 or 1, depending if an SCR occurred within the time frame. On rare occasions, a participant demonstrated two SCRs during the time frame, but it was still coded as “1.” The following equation was used to calculate each participant’s SCR score for Event and Non-Event videos:

$$SCR\;Score\left(\%\right)=\frac{no.\;ofclipswith\;SCR}{total\;no.\;ofclips}\times 100$$

This score represented the proportion of clips within each video type that elicited an SCR.

### Data Analysis

All analyses were conducted using IBM Statistics SPSS v26. To compare Learner and Licensed drivers on SCR score, a mixed model ANOVA was used with Group as the between subject variable and Video Type as the within subject factor. We reported main effects and interactions from this analysis. *Post hoc* pairwise comparisons were examined with Bonferroni corrections applied for multiple comparisons. Due to our small sample, when values of *p* approached significance, we calculated effect sizes. Effect sizes were assessed using Cohen’s *d* with small, medium, and large effect sizes as Cohen’s *d* 0.3–0.5, 0.5–0.8, and ≥0.8, respectively (Cohen, 1992).

Data were also examined for influences due to age, sex, and the task design effects of trial order and *Onset Time*.

## Results

### Participant Demographics

Participants included 41 drivers aged 16–20-years old. Three participants were excluded from analyses after reporting regular use of medications known to blunt SCR response. For the remaining 38 participants, 20 were Learner drivers who reported holding a United States learner’s permit for 1.29 ± 1.05 years (mean ± *SD*) and driving less than 900 miles of self-reported miles (mean ± *SD*: 293 ± 306). The Licensed drivers (*n* = 18) had a United States driver’s license (*n* = 16) or International license (*n* = 2) for at least 2 years (mean ± *SD*: 2.74 ± 0.62 years) and had more than 3,000 self-reported driving miles in the past 12 months (mean ± *SD*: 4,766 ± 1,708). All participants had normal or corrected to normal vision and did not report colorblindness.

The male:female ratio for the groups was: Licensed 11:7; Learner 6:14; [*χ*^2^(1) = 3.71, *p* = 0.054]. Licensed drivers were slightly older than Learner drivers, *F*(1,36) = 5.79, *p* = 0.021. We had no missing data. Licensed and Learner drivers differed significantly in number of miles driven, *F*(1,36) = 132.78, *p* < 0.001 such that Licensed drivers reported a higher number of miles driven compared to Learner drivers. Additionally, Licensed drivers reported having driven for more years than Learner driver, *F*(1,36) = 26.00, *p* < 0.001. Participant demographics are presented in Table 1.

**Table 1:** Demographics and driving history.

	Learner (*n* = 20)		Licensed (*n* = 18)		Group comparisons	
	Mean(*SD*)	Range	Mean(*SD*)	Range	*F*-statistic	*p* value
Age (years)	18.37(1.78)	16.12–20.87	19.47(0.79)	18.04–20.78	5.79	0.021
Sex^*^	6M/14F		11M/7F		3.71	0.054
Miles driven^**^	293 (306)	0–900	4,766(1708)	3,000–9,240	132.78	<0.001
Time driving^***^	1.29(1.05)	0.06-3.02	2.74(0.62)	2.02–4.17	26.00	<0.001

* Pearson Chi-Square value reported.

** Miles driven: self-reported total mileage for learner drivers; self-reported mileage in the past 12 months for licensed drivers.

*** Time driving: number of years from permit (for learner) or license (for licensed) issued date and time of study assessment.

### Analysis of SCR Score

Preliminary analyses revealed no significant main effects of age or sex, and no interaction with other variables; thus, we omitted sex from the model, but we included age as a covariate as there were group differences. There was no apparent video order effect, but a mixed model ANOVA (Onset Time: Early, Mid, and Late) × 2 (Event) × 2 (Group) revealed a significant three-way interaction [*F*(1,36) = 5.669, *p* = 0.023, *r*^2^ = 0.136, observed power 0.639] indicating group had a different effect on SCR score depending on Onset Time and Event Type.

#### Do Learner and Licensed Drivers Experience Differences in Psychophysiological Reactions to a Driving Hazard?

The results of a 2 × 2 mixed model ANOVA, with Video Type as the within subject factor (Event or Non-Event) and Group as the between subject factor (Learner or Licensed), revealed a Group effect that approached significance [*F*(1,36) = 3.623, *p* = 0.065, *r*^2^ = 0.094, observed power 0.457, *d* = 0.64]; the medium effect size indicating Licensed drivers were more likely to have an SCR response than Learner drivers (mean_Lic_ ± *SD* = 32.06 ± 16.18; mean_Learn_ ± *SD* = 21.71 ± 16.12). There was no significant interaction effect [*F*(1,36) = 0.474, *p* = 0.496, *r*^2^ = 0.013, observed power 0.103], suggesting that the group effect was consistent across Event and Non-Event videos.

**Figure 2:**
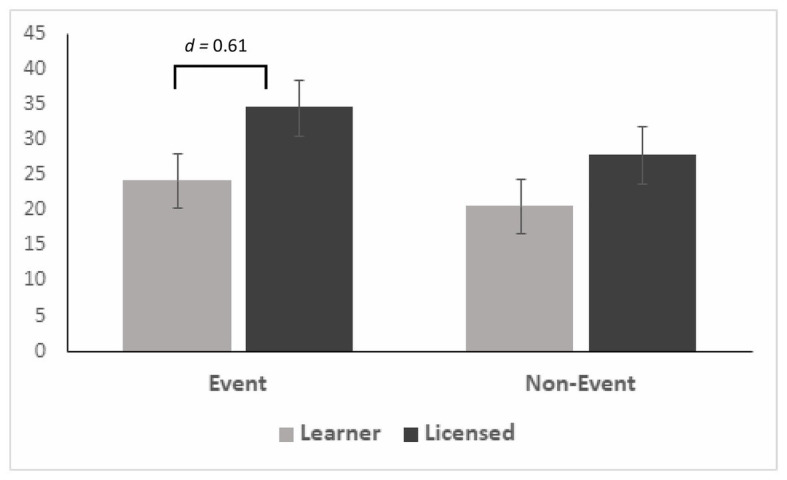
Mean skin conductance response (SCR) score by Group and Video Type with SE Bars.

**Figure 3:**
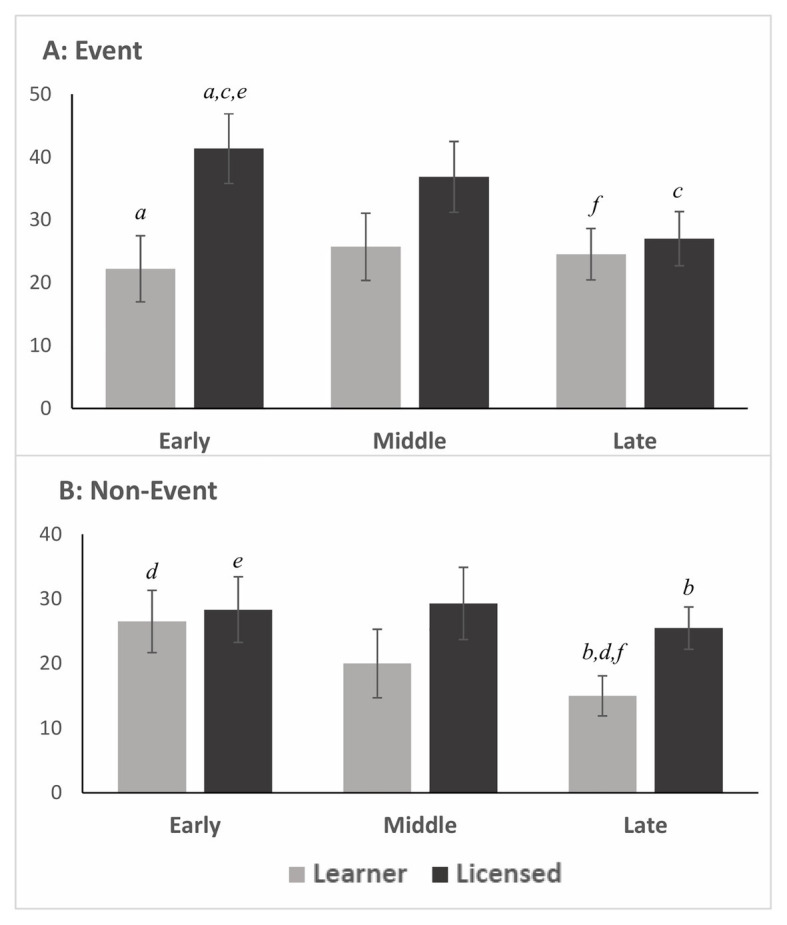
Skin conductance response score by Onset Time and Group. SE bars. Matched letters indicate statistically significant differences in mean score. Panel **(A)**: in the Event videos, **(a)** Licensed drivers had a greater SCR score in the *Early Onset* compared to Learner drivers; **(c)** Licensed drivers had a greater SCR score in *Early Onset* compared to *Late Onset*. Panel **(B)**: in the Non-Event videos, **(b)** Licensed drivers had a greater SCR score in the *Late Onset* compared to Learner drivers; **(d)** Learner drivers had a greater SCR score in the *Early Onset* compared to *Late Onset*. Across Panels: **(e)** Licensed drivers had a greater SCR score in the Event *Early Onset* compared to Non-Event *Early Onset*; **(f)** Learner drivers had a greater SCR score in the Event *Late Onset* compared to the Non-Event *Late Onset*.

Pairwise comparisons further probing the Group effect approached significance for the Event Videos [*F*(1,37) = 3.524, *p* = 0.069, *d* = 0.61], the medium effect indicating Licensed drivers more likely to have an SCR response than Learner drivers in the Event videos (mean_LicEvent_ ± *SD* = 34.59 ± 17.96; mean_LearnEvent_ ± *SD* = 24.24 ± 16.02; see Figure 2). The difference between groups did reach significance in the *Early Onset* Event Videos, [*F*(1,37) = 6.259, *p* = 0.017; mean_LearnEvent_ ± *SD* = 22.22 ± 18.73; mean_LicEvent_ ± *SD* = 41.36 ± 27.96] indicating a greater likelihood of an SCR in the Licensed drivers compared to Learner drivers if the hazard appeared early (Figure 3, letter a).

There was no Group effect for the Non-Event Videos [*F*(1,37) = 1.691, *p* = 0.202, *d* = 0.42], yet in the *Late Onset* Non-Event Videos, there was a significant difference between groups [*F*(1,37) = 05.393, *p* = 0.026; mean_LearnNonEvent_ ± *SD* = 15.00 ± 12.10; mean_LicNonEvent_ ± *SD* = 25.46 ± 15.60] indicating the Licensed drivers were more likely to have an SCR than Learner drivers during routine driving if the *measurement window* was toward the end of the video (Figure 3, letter b).

There was a significant difference between *Early* and *Late Onset* Event Videos in the Licensed group (mean_LicEarly_ ± *SD* = 41.36 ± 27.96; mean_LicLate_ ± *SD* = 27.02 ± 16.39; *p* = 0.024), indicating that the Licensed drivers were more likely to have an SCR response if the hazard developed earlier in the video compared to the end of the video (Figure 3, letter c). However, for the Learner group, there was a significant difference between *Early and Late Onset* Non-Event videos (mean_LearnEarly_ ± *SD* = 26.50 ± 20.33; mean_LearnLate_ ± *SD* = 15.00 ± 12.10; *p* = 0.018), indicating the Learner drivers were more likely to have an SCR response earlier in the Non-Event videos compared to the end of the Non-Event video (Figure 3, letter d).

#### Are Drivers More Likely to Show an SCR in Event Videos Compared to Non-Event Videos?

There was not a significant effect of Video Type [*F*(1,35) = 0.109, *p* = 0.744, *r*^2^ = 0.003, observed power 0.062, *d* = 0.29], indicating a similar likelihood across all videos to have an SCR response (mean_Event_ ± *SD* = 29.14 ± 17.54; mean_Non-Event_ ± *SD* = 24.08 ± 17.20).

Yet, in *Early Onset videos*, there was a significant difference between video type in the Licensed group [*F*(1,17) = 5.776, *p* = 0.028; mean_LicEvent_ ± *SD* = 41.36 ± 27.96; mean_LicNonEvent_ ± *SD* = 28.33 ± 22.82] indicating Licensed drivers were more likely to have an SCR when a hazard developed compared to routine driving (Figure 3, letter e) when the *measurement window* was at the beginning of the video.

In the *Late Onset* videos, there was a significant difference in the Learner group between video types [*F*(1,19) = 5.794, *p* = 0.026, *r*^2^ = 0.234, observed power = 0.627; mean_LearnEvent_ ± *SD* = 24.55 ± 19.81; mean_LearnNonEvent_ ± *SD* = 15.00 ± 12.10] indicating Learner driver were more likely to have an SCR when a hazard developed than compared to routine driving (Figure 3, letter f) when the *measurement window* was at the end of the video.

## Discussion

The purpose of this study was to examine differences in the autonomic responses (SCR) of Learner and Licensed drivers in response to video stimuli in the United States, replicating past studies performed in the United Kingdom. These psychophysiological differences will provide insights into how experience should be incorporated in the algorithms monitoring driver cognitive state in this increasingly automated driving environment. Overall, Learner drivers did demonstrate fewer SCRs than the Licensed drivers during The Driving Hazard Perception Task, and Event videos appeared to discriminate between Learner and Licensed drivers. The medium effect size suggests the relationship between driving group and SCR score in event videos was meaningful (*d* = 0.61) but the sample was underpowered to reach statistical significance. This finding is consistent with previous research examining differences in autonomic arousal between novice and experienced drivers (Kinnear et al., 2013) in a controlled setting. The addition of Non-Event videos to the task was novel, and Licensed drivers were more likely to exhibit an SCR in and Event video than Non-Event if the hazard developed early. Conversely, Learner drivers were more likely to demonstrate an SCR in the Event videos if the hazard developed late.

The design of this study differs from the experiment that it sought to replicate in one critical aspect, and this may explain underpowered results compared to the clear picture of greater autonomic responses in experienced drivers in Kinnear et al. (2013). The stimuli used for this study were derived from naturalistic driving dashcam footage as opposed to the professionally filmed hazard perception clips that were used in the United Kingdom study. The United Kingdom clips were developed during the design of the official hazard perception test and went through a detailed validation process (Grayson and Sexton, 2002). As such, they included validated examples of developing hazards and included defined timing windows, allowing differentiation between “anticipatory” (or precursory) and “event” areas in the time window of the defined hazard. Differentiation between the anticipatory and event periods could not be as clearly made in this study because the experimental stimuli lacked an extended build up period. This lack of definition between the anticipatory and the event stages of the hazards may have masked differences between novice and more experienced drivers and points to the importance of the experimental stimuli in measuring hazard perception.

This study also found an influence of *Onset Time* that had not been previously examined. This may also be an experiment artifact related to the specific stimuli, but the findings do suggest some important factors as it relates to autonomous driving and driver monitoring. Licensed drivers had a pattern of *decreased* likelihood of producing an SCR when the hazard developed later in the video, yet the Learner drivers maintained similar responses regardless of the timing of the hazard. This finding provides evidence that may be indicative of situational awareness as it relates to hazard *prediction*. As more experienced drivers have more time to observe the environmental and behavioral stimuli in a developing scene, they are better able to predict possible behaviors, and thus less likely to elicit an SCR when a predicted hazard occurs. Learners, on the other hand, were equally “surprised” when the hazard developed regardless of the time spent observing the situation. This theory coincides with current work indicating hazard prediction is the subcomponent of hazard perception that differentiates experienced and novice drivers (Crundall, 2016; Ventsislavova et al., 2019). The similar pattern of *decreased* likelihood of producing an SCR later in the video was observed in the Learner drivers in the Non-Event videos. The Licensed drivers, in contrast, had a consistent likelihood of an autonomic response regardless of the timing of the *measurement window*. We have labeled these videos as Non-Event, but the routine driving captured by the dashcam will inherently include situational cues to which more experienced drivers may respond. While the Licensed drivers consistently attend to these potential hazard cues throughout an “uneventful” video clip, the patterns observed in the Learner drivers may be physiological evidence of previously identified novice deficiencies in lack of awareness and sustained vigilance.

This interpretation of differences in situational awareness and sustained vigilance is presented with caution for there are other possible interpretations. While there was an overall lower reactivity of Learner drivers, it may be these drivers needed a longer period to discriminate between Event and Non-Event videos, and thus video type differences were only seen in the *Late Onset* videos for this group due to sustained vigilance. In contrast, the Licensed driver decreased reactivity in Event videos as time progresses may be indicative of decreased sensitivity rather than prediction. Regardless, the timing of the *measurement window* in a naturalistic driving video does need to be investigated as it points to different levels of experience may predispose drivers to hazard detection vulnerabilities that manifest at different stages on the driving task. These investigations should include several task versions with different timing windows for the same stimuli. This research design would clearly examine the influence of timing independent from possible stimuli specific responses that is a limitation of the current study. Additionally, this would improve the input to driving monitoring algorithms determining the appropriate wait time between driver hazard orientation and expected SCR.

While this study describes our groups as differing in experience, exposure and experience are not the same thing. An individual driving on the same routes is not likely to be as experienced as an individual who is driving on different road types and in varied traffic scenarios. However, epidemiological evidence suggests that exposure and experience (and crash risk) are related (Elvik, 2006). As there is no established measure of experience, participants were screened for inclusion in the study based on their exposure as a proxy of experience. Additionally, there was a significant difference in age between the two groups, as well as a lower age range of the Learner group, but age was used as covariate in the analyses.

In conclusion, this experimental study used a novel Driving Hazard Perception Task and measured SCR during videos where a hazard occurred (Event), and videos of routine driving (Non-Event). A medium effect size suggests that videos containing a hazard (Event) appeared to discriminate between novice and more experienced drivers, but the sample was underpowered to reach statistical significance. The confounding influence of *Onset Time* may be an additional factor influencing the findings. The decreased likelihood of SCR as the videos progress may be an indicator of situational awareness that needs further investigation. While physiological measures such as SCR may be useful for research and real-time state measurement relating to automated technologies and situational awareness, more needs to be understood with regard to experimental design and use of stimuli for validating such standards. Regardless, our research provides evidence that SCRs relative to hazard perception do differ based on experience, and this needs to be included in the model of driver state monitoring to properly understand the physiological signal and to facilitate safe integration of ADAS technologies in vehicles.

## Data Availability Statement

The raw data supporting the conclusions of this article will be made available by the authors, without undue reservation, to any qualified researcher.

## Ethics Statement

The studies involving human participants were reviewed and approved by Johns Hopkins Medicine Institutional Review Board (IRB# 170352). Written informed consent to participate in this study was provided by the participants’ legal guardian/next of kin.

## Author Contributions

TC wrote the manuscript and was responsible for data collection, analysis, and interpretation. TC also assisted in the development of the novel driving hazard perception task. JE assisted in the writing of the manuscript, data interpretation, and was responsible for the research concept. NK assisted in the writing of the manuscript, data interpretation, and laid the foundational work for this paper. KS assisted in the writing of the manuscript, data interpretation, led the development of the driving hazard perception task, and was responsible for the research concept. All authors contributed to the article and approved the submitted version.

### Disclaimer

This work was prepared while KS was employed at Johns Hopkins University and Kennedy Krieger Institute. The opinions expressed in this article are the author’s own and do not reflect the view of the National Institutes of Health, the Department of Health and Human Services, or the United States government.

### Conflict of Interest

The authors declare that the research was conducted in the absence of any commercial or financial relationships that could be construed as a potential conflict of interest.
