# Supplementary material for: Skin Conductance Responses of Learner and Licensed Drivers During a Hazard Perception Task
Source: Front Psychol. 2021 Mar 25;12:619104. doi: 10.3389/fpsyg.2021.619104 (PMC8026887; doi:10.3389/fpsyg.2021.619104)
Supplement: Supplementary file 1 [file Table_1.docx]

**Supplemental Materials**

**S.1 Driving Hazard Perception Task**

The development of the hazard prediction task is reported in Ehsani et al. (2020). Briefly, 30 videos were selected from the Strategic Highway Research Program (SHRP 2) naturalistic driving dataset from the Naturalistic Driving Study (NDS) (National Academies of Science Engineering and Medicine, 2012). Selected videos met the following criteria: (1) Near-crashes with an ‘event start and event end’ duration of four seconds or longer, (2) No apparent driver impairment which mean the hazards were due to actions of other road users, and (3) Met a pre-defined driving conflict type (lead vehicles, oncoming traffic, vehicles in an adjacent lane, merging vehicles, turn into traffic, turning across traffic, at intersection, with parked vehicle, pedestrians, cyclists and with objects in road). All selected videos were in the daylight and non-hazardous weather conditions.

Videos provided by the Virginia Tech Transportation Institute as part of their data support for the SHRP2 study were 90 seconds long with the near crash event (i.e. the moment the driver needed to take evasive action) centered at the 45 second mark. These videos were edited to create a 30 second portion with the near-crash event (identified as *Event videos*) and a 30 second portion from the same video that included routine driving (identified as *Non-Event videos*) without any hazardous events requiring a response from the drivers (i.e. braking or swerving).

The occurrence of the near-crash event in the Event videos were staggered to avoid learning effects. That is, the near-crash event occurred in the first 10 seconds for 10 videos (classified as Early Onset), between 10-20 seconds for 10 videos (classified as Middle Onset), and in the final 10 seconds for 10 videos (classified as Late Onset). The *measurement window,* approximating the *anticipatory period* from the Kinnear et al. (2013) study, was defined from the first frame the hazard appeared on the screen to 3 seconds after the driver was required to perform an evasive action. In Non-Event videos, non-hazardous occurrences were randomly selected from general driving clips with timing that corresponded to the event videos and were also staggered to have Early, Middle, and Late Onsets. **Table 1S** includes descriptions of the videos and the events defining the *measurement window*.

The final collection of 60 videos were randomized and presented to all participants in the same order. The Driving Hazard Prediction Task was programmed in Presentation® software (Version 18.1, Neurobehavioral Systems, Inc., Berkeley, CA, [www.neurobs.com](http://www.neurobs.com)). To ensure attention during the task, participants were asked to rate “How likely was the driver of this car to get into a crash?” after each video. Analyses of the rating data can be found in Ehsani et al. (2020).

**S.2. Study Visit**

Participants were seated in a chair at a curved laboratory table allowing for the non-dominant hand and arm to rest comfortably on cushioning to minimize movement. Two electrodes were placed on the distal phalanx of the index and middle finger of the non-dominant hand to measure skin conductance, and participants were asked to rest with the palm facing up and to keep the hand still during the task. The electrodermal activity measurements were performed by means of a physiological signals acquisition unit (MP160, Biopac Systems Inc., Santa Barbara, CA).  The Driving Hazard Perception Task was viewed on a laptop computer screen, 41.5cm x 27.9cm, perpendicular to the line of sight. Participants moved the laptop to a comfortable viewing distance, an average of 60 cm from their eyes. Physiological recordings were recorded on a separate laptop computer.

After a 2-minute baseline period, the experimenter began the task by showing the instructions on the task screen and reading them aloud.

Screen 1: “Welcome to the Driving Hazard Perception Task. In this task, you will be shown several driving videos. Each video was recorded from a camera mounted on the windshield of a car, so that you have the viewpoint of the driver. Please watch each video carefully. At the end of each video, you will be asked to rate how likely was the driver of this car to get into a crash. 1 means not likely to 5 meaning very likely [Likert Scale shown on the screen]. The task will last about 30 minutes. Do you have any questions?”

Screen 2: “Before we begin the actual task, we are going to do some practice trials. At the end of each video, you will be asked, “How likely was the driver of this car to get into a crash?” [Likert Scale shown again]. Use the right and left arrow keys on the keyboard to select your answer and press ENTER when you are done. Are you ready to get started with the practice trials?”

Participants were also instructed that if they needed to take a break or move the equipment hand, they may do so once a video ended and prior to selecting their rating as the task did not move on to the next video until the participant moved the curser to the desired rating and pressed enter.  The practice session lasted approximately 4 minutes, and the task lasted approximately 35 minutes (30 minutes of videos and an estimated 5 seconds for each rating).

**Table 1S: Description of Videos and Frames Identified for *Measurement Windows***

| **SHRP2 Classifications** | | | **Event Video** | | **Non-Event Video** | |
| --- | --- | --- | --- | --- | --- | --- |
| **Video ID** | **Conflict With** | **Locality** | **Hazard Event Start** | **Critical Moment** | **Neutral Event Start** | **Neutral Moment** |
| 10596833 | Merging vehicle | Business/  Industrial | Merging vehicle is visible | Visible deceleration by driver | Non conflict car visible | Passing car visible |
| 29858001 | Vehicle in adjacent lane | Interstate/  Bypass/Divided Highway | Driver begins to change lanes. | Visible swerve by driver | Approach turn only lane | Approach left exit road |
| 29858020 | Merging vehicle | School | Merge is visible | Visible swerve by driver | Driver enters merge lane | Driver begins to pass truck in adjacent lane |
| 29878392 | Vehicle turning across another vehicle path (same direction) | Interstate/  Bypass/Divided Highway | Truck slowing down for unknown reason. | Visible deceleration and swerve by driver | Driver begins passing car in adjacent lane | Side road visible |
| 30883594 | Vehicle turning into another vehicle path (opposite direction) | Business/  Industrial | T-intersection visible | Visible deceleration and swerve by driver | Approaching car appears | Approaching car passes |
| 31297598 | Vehicle in adjacent lane | Interstate/  Bypass/Divided Highway | Lead car signals to change lanes | Visible swerve by driver | Enter shadow under bridge | Driver passes overhead sign |
| 33575108 | Vehicle in adjacent lane | Interstate/  Bypass/Divided Highway | See multiple cars with break lights | Visible swerve by driver | Driver passes slowed truck | Visible car changes lanes |
| 51722141 | Vehicle turning across another vehicle path (same direction) | Moderate Residential | Van is first visible on side street | Visible swerve by driver | Traffic light clearly visible | Driver begins to turn |
| 128906312 | Vehicle turning across another vehicle path (same direction) | Urban | Conflict vehicle is visible | Visible deceleration and swerve by driver | T-intersection becomes visible | Car in opposite direction passes driver |
| 135366668 | Vehicle turning across another vehicle path (same direction) | Interstate/  Bypass/Divided Highway | Conflict vehicle is visible | Visible swerve by driver | Green traffic light visible | Driver passes under traffic light |
| 142048318 | Pedestrian | Moderate Residential | First see pedestrian on side of road | Visible deceleration by driver | Driver passes stoplights | Driver reaches median |
| 144804951 | Vehicle turning into another vehicle path (opposite direction) | Business/  Industrial | Conflict vehicle is visible | Visible swerve by driver | Approaching car appears | Last approaching car in a line passes |
| 145481265 | Vehicle turning across another vehicle path (same direction) | Open Residential | Conflict vehicle is visible | Visible deceleration and swerve by driver | Car at stoplight visible | Driver begins to accelerate after light turns green |
| 150716768 | Vehicle turning across another vehicle path (same direction) | Interstate/  Bypass/Divided Highway | Conflict vehicle is visible | Visible swerve by driver | Driver passes trees in a median | Driver reaches end of trees in median |
| 150717124 | Pedestrian | Interstate/  Bypass/Divided Highway | First see pedestrian on side of road | Visible deceleration by driver | Non conflict vehicle begins to move | Driver approaches van |
| 151568478 | Merging vehicle | Interstate/  Bypass/Divided Highway | Merging vehicle is visible | Visible deceleration by driver | Driver passes overhead road sign | Guardrails visible |
| 151568780 | Lead vehicle | Moderate Residential | See car merging at start of video | Visible deceleration by driver | Driver comes out from shadow | Driver passes road sign |
| 151569062 | Merging vehicle | Interstate/  Bypass/Divided Highway | Merging vehicle is visible | Visible deceleration by driver | Exiting cars visible | Driver passes exiting cars. |
| 151569269 | Vehicle in adjacent lane | Interstate/  Bypass/Divided Highway | Adjacent vehicle begins to switch lanes (blinkers start). | Visible deceleration by driver | Driver passes under overhead bridge; | Driver passes under road signs |
| 151570615 | Vehicle in adjacent lane | Business/  Industrial | Conflict vehicle begins to cross lanes. | Visible deceleration by driver | Driver begins to pass car | Driver finishes passing car |
| 151573899 | Merging vehicle | Urban | Merging vehicle is visible | Visible swerve by driver | Driver passes highway sign | Driver passes exit sign |
| 151586128 | Merging vehicle | Bypass/Divided Highway with traffic signals | Car parked on shoulder becomes visible. | Visible swerve by driver | Diver begins navigating turn | Driver finishes turn |
| 151586135 | Vehicle in adjacent lane | Interstate/  Bypass/Divided Highway | Truck and merge present at beginning. | Visible deceleration of driver. | Non conflict vehicle starts to turn | Traffic light turns green |
| 151586560 | Merging vehicle | Interstate/  Bypass/Divided Highway | Merge is visible | Visible deceleration of driver. | Driver passes overhead road sign | Driver passes another overhead road sign |
| 151591696 | Lead vehicle | Interstate/  Bypass/Divided Highway | Lead vehicle starts to break. | Visible deceleration of driver. | Visible car's break lights turn off | Visible car has greatest acceleration |
| 151593427 | Merging vehicle | Interstate/  Bypass/Divided Highway | Truck parked on roadside if visible | Visible deceleration of driver. | Parked vehicle visible | Driver passes parked vehicle |
| 151606560 | Vehicle in adjacent lane | Interstate/  Bypass/Divided Highway | Merge and car visible from first frame. | Visible deceleration of driver. | Driver begins the ascent of the bridge | Driver reaches the decent of the bridge |
| 151859779 | Merging vehicle | Business/  Industrial | Parked car and truck in scene with narrowing lanes. | Visible deceleration and swerve by driver | Adjacent car on left emerges into view | Driver begins to pass truck |
| 151859877 | Merging vehicle | Church | Conflict vehicle begins to merge | Visible deceleration by driver | Driver passes blinking arrow | Driver enters between toll booth guide rails |
| 151859975 | Lead vehicle | Interstate/  Bypass/Divided Highway | Stationary object in front of lead vehicle visible | Visible deceleration by driver | Visible car commences lane change | Visible car completes lane change |

Ehsani, J. P., Seymour, K. E., Chirles, T., & Kinnear, N. (2020). Developing and testing a hazard prediction task for novice drivers: A novel application of naturalistic driving videos. *Journal of Safety Research*, *73*, 303–309. https://doi.org/10.1016/j.jsr.2020.03.010

Kinnear, N., Kelly, S. W., Stradling, S., & Thomson, J. (2013). Understanding how drivers learn to anticipate risk on the road: A laboratory experiment of affective anticipation of road hazards. *Accid Anal Prev*, *50*, 1025–1033. https://doi.org/10.1016/j.aap.2012.08.008

National Academies of Science Engineering and Medicine. (2012). *The SHRP2 Naturalistic Driving Study (NDS)*. https://insight.shrp2nds.us/documents/shrp2_background.pdf
